# Digitalization's contribution towards sustainable development and climate change mitigation: An empirical evidence from EU economies

**DOI:** 10.1016/j.heliyon.2024.e33451

**Published:** 2024-06-24

**Authors:** Zeeshan Arshad, Mara Madaleno, Ana I. Lillebø, Helena Vieira

**Affiliations:** a CESAM – Centre for Environmental and Marine Studies, Department of Environment and Planning, University of Aveiro, Aveiro, Portugal; b GOVCOPP - Research Unit on Governance, Competitiveness and Public Policy, DEGEIT - Department of Economics, Management, Industrial Engineering and Tourism, University of Aveiro, Campus Universitario de Santiago, 3810-193, Aveiro, Portugal; c ECOMARE, CESAM-Centre for Environmental and Marine Studies, Department of Biology, Santiago University Campus, University of Aveiro, 3810-193, Aveiro, Portugal

**Keywords:** Climate change mitigation, Digitalization, Econometric, Machine learning, Sustainable development

## Abstract

The current study aims to test the usage of econometric and machine learning approaches to study the relationship between methane (CH_4_), a hydrocarbon component of natural gas, as a proxy of carbon emission, GDP as economic growth, financial development (FIN), and medium and high technologies as a proxy of information technology (ICT) and human development (HDI). This study observes two extended moderating effect models of human development index and financial development via medium and high technologies on carbon emissions over the 15-year periods from 2007 to 2021 for the 27 EU economies. Results indicate that when considered solely, ICT, economic growth, and HDI improve environmental quality and contribute to climate change mitigation, reducing methane emissions, whereas financial development seems to damage environmental quality. However, the crossed effects of ICT with HDI, and that of ICT with FIN, were considered in estimations, with results pointing out that those favorably affect climate change mitigation. Jointly considering ICT, HDI, and financial development proves to have a synergistic effect in promoting environmental health than each element on its own. Green and yellow countries were also identified revealing the countries for which a reduction and increase, respectively, in the value of methane emissions is predicted after three years. In the case of the entire panel, the STR (linear regression tree) algorithm predicts an average growth in methane emissions of around 3.64 %. Important policy directions are drawn considering the results obtained.

## 1 Introduction

Recently, climate change needs, sustainability requirements, local and global conflicts, consumers' and producers' awareness, and legal impositions have forced countries to redirect their priorities to a more sustainable growth pattern [1]. With the urgent and intensified call for reducing greenhouse emissions, research has turned attention to information technology (ICT) and the role of digitalization in curbing the effects of climate change, considering these are both crucial tools for both a more efficient use and cleaner production of energy [2].

Besides the clear role of digitalization in climate change mitigation and sustainable development, Nguea [3] found that trade openness, financial development, electricity consumption, and democracy are the main channels through which ICTs positively improve human development, at least in Africa. Moreover, considering that clean energy productions are mandatory for sustainable development [4], and similarly critical for technology advancements such as renewables and ICTs, their enhancement also leads to human development [5]. Additionally, Nkemgha et al. [6] analyze the effect of infrastructures on industrialization, through financial development and human capital channels, in 33 African countries, evidencing that infrastructural development has, as expected, a direct enhancing effect on industrialization in Africa. In that context, access to financial sources is crucial, which is only possible with a prosperous financial system development. Moreover, Saqib et al. [7] examine the relationship between economic growth, financial development, ICT, renewable energy, and human capital with carbon footprint in the case of world-polluting economies. Authors noted that ICT has the potential to impact positively on carbon emissions.

Adding to this, Islam et al. [8] examine the asymmetric nexus among ICT, energy use, financial development, and CO_2_ emissions in GCC countries. The authors employ 2nd generation unit root and Westerlund cointegration tests, and panel-pooled “generalized least square (GLS)” techniques. The “pooled mean group (PMG)” method and the “Dumitrescu-Hurlin (D-H)” causality test are then employed to verify the robustness of GLS estimation. Results exhibit an asymmetric relationship between ICT and CO_2_ emissions, noticing that ICT and financial development reduce emissions, validating the cointegration nexus among ICT-energy-financial development- CO_2_ emissions. Using data from 36 countries, Ngo et al. [9] report a bi-directional nexus between financial development and green growth, with human capital and education expenditure appearing at the forefront of sustainable development. However, not all literature suggests a positive role in financial development. Verma et al. [10] found that ICT diffusion favors sustainable human development, as does globalization and economic growth. However, the impact of government effectiveness and financial development was found to be insignificant. Similarly, Ben Jebli et al. [11] noticed that ICT and renewable energy improve the environmental quality in 84 countries around the Globe.

Digitalization is a crucial facilitator of the socioeconomic dynamics and sustainability of cities [12] as well as of countries [13], with the ability to build climate-friendly landscapes and communities. With the arrival of the fourth industrial revolution, there has been a greater use of digitalization in a variety of industries and at various levels. High-tech digital gadgets, platforms, and settings are rapidly being used to boost productivity, efficiency, and sustainability, as well as to improve people's general well-being [6,12]. Digitalization is expected to have a greater influence on regions in the future, transforming occupations and causing lifestyle changes with far-reaching consequences that will eventually damage regions' resilience and adaptive capacities. While a growing corpus of research has emphasized the importance of digitalization in climate change mitigation, such as lowering GHG and CO_2_ emissions, thorough assessments of digitalization's potential as an aid to climate change mitigation are lacking [2,12]. The literature also recognizes that economic growth, foreign direct investment, and trade openness contribute positively to the ecological footprint. But whereas these factors are found to worsen environmental quality, urbanization, financial development, natural resources, and human capital are found to decrease ecological footprint, thus contributing to effective climate change [14].

The contribution of the article is thus threefold. First, there is still no consensus in the literature as to the effects of these macroeconomic variables on climate change mitigation, nor enough studies realizing the real impacts of interaction terms over emission levels, especially considering methane emissions. With these gaps in mind, the current research work aims to identify the role of macroeconomic variables such as information technology (ICT), Financial development (FIN), Human development (HDI), and economic growth (GDP) in climate change mitigation. Moreover, the study also explores the moderating role of ICT in human development, and financial development on methane emissions intensity, by considering the cross effects of these variables in the equation. The results obtained allowed the following contributions to the field. First, although current research has offered prolific information to uncover the role of HDI, financial development, ICT, and economic growth on environmental sustainability, relevant studies on assessing the moderating effect of ICT on HDI and Financial development in EU countries are scarce.

Second, the current study uses the Natural Logarithm of Medium and High-technology exports (% of manufactured exports) as a proxy of ICT and Methane (CH_4_) as well as Carbon Dioxide (CO_2_) as emissions indicators. Carbon emissions dominate when we talk about human-made greenhouse gases [15]. But CO_2_ is only part of the total GHG emissions (greenhouse gas (GHG) emissions include CO_2_ (carbon), CH4 (methane), and N2O (nitrous oxide) emissions). The amount from industry and energy hit a whopping 535 billion tons of CO_2_ emissions. However, methane and nitrous oxide should not be overlooked, considering they are doing their part in heating the planet too (global warming), especially between 2014 and 2016. About a third of the emissions from the past ten years come from these two types of emissions, thanks to tropical biomass and burning fossil fuels in cooler regions [8]. Still, most of the literature when accounting for climate change measures concentrates their analysis on CO_2_ emissions. Solarin et al. [16] called attention to the fact that the overlooked greenhouse gas methane was seldom addressed. This study's main goal is to see if methane emissions in 37 OECD (Organisation for Economic Co-operation and Development) countries stochastically converge over 200+ years. Results from customary unit root tests and a new wavelet unit root test with a Fourier function strongly show these countries' methane emissions diverge.

Third, one of the crucial gaps is that prior studies ignored the machine learning process of regression algorithms and the best-fitted method used to forecast each sampled country's methane. The current study aims to develop the relationship amid methane (CH4), a hydrocarbon component of natural gas, as a proxy of emissions (climate change), GDP as economic growth, financial development (FIN), and medium and high technologies as a proxy of information technology (ICT) and human development (HDI).

To perform this analysis a literature review considering the main interactions among macroeconomic variables was performed (Section 2). Section 3 presents the data and methodologies applied for the presentation of the results which are available in Section 4. Section 5 discusses the main policy implications derived from the results, whereas Section 6 concludes this work. Despite traditional panel data methodologies implementation, after confirming the cointegration among the variables, through the dynamic auto-regressive distribution, the lag-cross-sectional (ARDL-CS) technique was jointly combined with machine learning methods allowing the assessment of both the group of green and yellow countries, which respectively represent the countries for which a reduction and increase, in the value of methane emissions is predicted after three years. Finally, this study observes two extended moderating effect models of the human development index and financial development via medium and high technologies on carbon emissions over the 15-year periods from 2007 to 2021 for the 27 EU economies. Based on the findings of this article important policy implications are derived for the sample of 27 EU countries.

## 2 Literature review

The connection between macroeconomic variables and environmental sustainability has received significant attention in the academic discourse. Several studies have discussed the relationship among macroeconomic variables, spotted the impact on carbon emissions in the existing literature, and obtained a diversified outcome. In addition, prior scholars noted the positive and negative impacts of the blend of different macroeconomic variables on carbon emissions. Nonetheless, the current research work aims to identify the role of macroeconomic variables such as information technology (ICT), Financial development (FIN), Human development (HDI), and economic growth (GDP) in climate change mitigation. Moreover, the study also explores the moderating role of ICT in human development and financial development on carbon emissions intensity (using methane as a proxy). The following section of the study discusses the nexus between the different considered macroeconomic variables to highlight the theoretical and empirical relationship in the existing literature.

### 2.1 Economic growth and carbon emissions nexus

Over the last couple of decades, the most common discussion among researchers, policymakers, and stakeholders has been climatic change, environmental issues, sustainability of natural resources, and other upcoming food-related issues around the Globe. The main objective was to address the climate problems and ensure the sustainability of natural resources to achieve the United Nations' goals and objectives by 2030 [17]. Moreover, stakeholders are also focusing on finding other protein sources to fulfill the need for upcoming food demand and employment opportunities for all, with the progression of the population around the planet. In several studies, the most commonly used economic indicators are economic growth and carbon emissions to identify the impact of climate change. However, those studies addressed this concept with the different proxies of Greenhouse Gas (GHG) emissions such as CO_2_ emissions per capita, methane, carbon footprints, sulfur dioxide (SO_2_), sulfur hexafluoride (SF_6_), perfluorocarbons (PFCs), hydrofluorocarbons (HFCs) and particulate matter PM2.5, and PM10 as air pollutants.

The relationship between economic growth and carbon emissions is known as the Environmental Kuznets Curve (EKC). The theoretical definition of EKC claims the inverted U-shaped relationship between carbon emissions and economic growth. It means that in the early stage, carbon emissions grow with economic progression, and after reaching a certain threshold level, it goes down [18,19]. However, in the existing literature, studies found mixed empirical findings with some of them finding the validation of the EKC, and others not. Considering those that validate the EKC hypothesis, for instance, Sterpu et al. [20] did it for the case of 17 out of 28 European nations from 1990 to 2016. Bekun et al. [21] with the method of the Mean Group (MG), Augmented Mean Group (AMG), Common Correlated Effects Mean Group (CCMG), and Dumitrescu and Hurlin (DH) causality for 27 EU countries also validate the EKC hypothesis. Balsalobre et al. [22] for 5 EU countries with panel cointegration and regression methods throughout 1990–2015, Saqib et al. [23] for 22 EU nations, Balsalobre-Lorent et al. [24] for 5 EU countries from 1990 to 2017, Magazzino et al. [25], Leitão et al. [26] and Jahangir et al. [27] for 27 EU, 4 EU and the top ten nuclear countries with panel ARDL, respectively, all validate the EKC. Moreover, Feng et al. [28] for China, Xing et al. [29] for Asian economies, Mahmood et al. [30] for the Latin countries throughout 1970–2019, Saia [31] for 55 countries with the PSTR approach, Bao and Lu [32] for the 27 EU economies, Wang et al. [33] for 208 countries around the world throughout 1990–2018, Kostakis et al. [34] for the MENA countries, and more recent studies in 2024 by Hameed et al. [35] for Afghanistan, and Li et al. [36] for 38 countries, all confirmed the validation of the EKC hypothesis.

Nevertheless, it is noteworthy that a subset of studies fails to align with the EKC hypothesis, potentially attributable to variations in econometric methodologies, temporal scopes, socio-economic milieus, and the choice of macroeconomic indicators [37]. In those unable to validate the EKC hypothesis we include, Pilatowska et al. [38] for Spain throughout 1970–2018, Dogan and Inglesi-Lotz [39] for the EU, Ketenci [40] for 15 EU countries throughout 1960–2015 with DOLS and cointegration approach, Madaleno and Moutinho [41] for 27 EU countries, Frodyma et al. [42] for 27 EU nations during 1990–2017 with ARDL bound testing approach, Tabash et al. [43] for 6 GCC nations for 2001–2019 with the FMOLS approach, Feng et al. [28] for China, Wang et al. [33] for 56 countries with AMG and Panel threshold regression, Hossain et al. [44] for India, Zhang et al. [45] for China, and Ciarlantini et al. [46] for 5 EU economies. Moreover, in 2024 we may also find some studies with unsuccessful validation of the EKC hypothesis such as Wang et al. [47] for the G20 economies and Nathaniel et al. [48] for developing economies.

### 2.2 ICT and carbon emissions degradation nexus

Several studies have discussed the influence of information technology on the degradation and improvement of environmental quality. For instance, Pan et al. [49] noticed that internet development improves the ecological quality of Chinese urban areas by upgrading industrial structures, promoting green innovation, strengthening environmental regulation, and promoting low-carbon development of resource-based cities. Furthermore, Islam and Rahaman [50] examined the effects of ICT on CO_2_ emissions in the case of Gulf Cooperation Council (GCC) countries throughout 1995–2019. The study employed the second-generation Westerlund cointegration and PMG technique to identify the long coefficients, and the DH causality test to check the causal direction between the considered variables. The authors confirmed the validation of the EKC and the existence of long-run relationships among the variables. In addition, the study found several bidirectional and one-way direction causalities running within the variables. The authors argued that promoting ICT is a critical model to improve the environmental quality in GCC regions. In another recent study, Jananger et al. [27] scrutinized the shape of the EKC hypothesis for the case of nuclear economies from 1990 to 2017. The authors alleged that nuclear energy, with the penetration of ICT, can be used to achieve sustainable economic growth. Moreover, Shobande and Asongu [51] noticed that 90 % of carbon emissions are linked to ICT production, installation, and usage in Africa. However, authors found mixed results, such as ICT contributing to South Africa's environmental sustainability, whereas in Nigeria, they noted the opposite. Moreover, Cui et al. [52] accomplished the future environmental effects of various kinds of ICT investment in China. The authors noted that using ICT software improved the environmental quality, whereas ICT hardware deteriorated the climate. In addition, ICT PCS has conducive effects on the environment, whereas ICT PCS has indirectly affected the environment through the digital economy and energy efficiencies. However, the improved environment can be achieved through optimizing ICT investments. Besides, Kwakwa et al. [53] evaluated the effect of urbanization, fertilizer usage, foreign direct investment, and ICT development on carbon emissions. They suggested that increasing ICT development can provide better environmental quality in Ghana. In addition, Zheng et al. [54] confirmed that ICT asymmetrically impacts carbon neutrality and sustainable growth.

Furthermore, Sun et al. [55] examined the role of ICT in carbon emissions in the case of high-income, middle, and low-income countries. The authors suggested that ICT development, especially in middle-income countries, allows better routes to achieve sustainable economic growth. Besides, Tsimisaraka et al. [56] also suggested that developing ICT infrastructure can promote a sustainable environment for the top ten emitter countries of OBOR. Moreover, Tzeremes et al. [57] claimed that ICT is a significant choice in developing energy transition and solving some environmental challenges for BRICS economies. Recently You et al. [58] noticed that ICT, human capital, and energy consumption improved the climate, whereas economic growth deteriorated the environment in the 64 Belt and Road initiative economies. Moreover, the authors also point out that the inverted U-shape exists between ICT and CO_2_ emissions. In another study, Uddin et al. [59] analyzed the impact of energy consumption, military spending, and ICT on CO_2_ emissions for the G20 countries covering the period of 1980–2019 with GMM and ARDL panel data approaches. Authors noted that higher military spending, energy consumption, and ICT worsened the environmental quality.

### 2.3 HDI and carbon emissions degradation nexus

The social inclusiveness is one of the significant predictors of carbon emission [60]. Manifold studies have proven that human development has mixed effects on the environment quality considering different periods, regions, and models around the Globe. However, high-income countries are more developed and keener to mitigate carbon emissions than low- and middle-income countries. If high-income nations allocate their resources to opportunities in low- and middle-income nations, their efforts to reduce climate change may have a more substantial impact [61].

Numerous studies analyzed the impact of human development on carbon emissions in the existing literature. For example, Liu et al. [62] examined the relationship between HDI, infrastructure, and CO_2_ emissions in China throughout 1990–2021. Authors noted that infrastructure played a vital role in the development of China, but in the same period, it also increased environmental pollution. Therefore, studies claim that green projects such as low-carbon transportation can improve the environmental conditions in China. In another study, Chen et al. [63] also discussed the coupling effect of energy-related carbon emissions and HDI in China. Studies argue that HDI is increasing; however, lessening energy-related carbon emissions is critical to achieving carbon neutrality in China. Moreover, Banday and Kocoglu [64] investigated the impact of HDI, energy consumption, and economic growth on carbon emissions for emerging economies between 1990 and 2014. The study noticed, with panel quantile regression, that a negative bilateral relationship exists between HDI and emissions. Furthermore, Bashir et al. [65] suggested a sustainable environment could be achieved through human development, natural resource rents, and energy evolution in OECD countries. In addition, Aqib and Zaman [66] also indicated that green development strategies can be used to achieve a sustainable environment in Pakistan. In another study, Sachan et al. [67] explored the relationship between environmental policy stringency, HDI, and renewable energy consumption for BRICS countries. Authors noticed that HDI and environmental policy stringency index positively impact ecological quality. However, the analysis suggested that promoting renewable energy consumption can be helpful to achieve sustainable goals. Recently, Leitão et al. [68] tried to identify the relationship among human development, carbon emissions, foreign direct investment, international trade, and renewable energy with quantile regression for G7 economies. The study noted that human development improved environmental conditions.

### 2.4 Financial development and carbon emissions degradation nexus

Financial systems are alleged to be an effective means of managing carbon emissions through efficient resource allocation [69]. Researchers and stakeholders have been closely monitoring the relationship between carbon emissions and financial development since the global economic crisis [70], and some believe that financial development can help reduce carbon emissions, while others argue the opposite. For example, Sunday Adebayo et al. [70] explored the relationship between financial development and carbon emissions for the MINT (Mexico, Turkey, Indonesia, and Nigeria) from 1969 to 2019. The authors noted that there is a causal association between financial development and CO_2_ emissions. In another study, Mar’I et al. [71] discussed the crucial role of financial development in addressing sustainable environmental challenges while considering the five highly polluted countries in the world. Authors noticed mixed results; for instance, in India, studies found positive and negative shocks while only negative shocks in China, Russia, USA, and Japan with quantile-on-quantile and nonparametric causality-in-quantile methodologies. The authors recommended that the embracement of sustainable financial practices helps to achieve a sustainable environment. Moreover, Ren et al. [69] investigated the role of financial development on carbon emissions from 2000 to 2019 in 30 provinces of China. Authors identified the reduction effect of carbon emissions when poverty levels are lower. Moreover, structural effects partially explain the negative relationship between financial development and emissions. In addition, the study argued that financial development improved the environmental quality at the regional level but deteriorated the environment in the neighboring regions. Likewise, Wiredu et al. [72] scrutinized the long-run relationship among HDI, financial development, economic development, and carbon emissions for African economies by using panel data from 1990 to 2020 with the ARDL-CS approach. The authors noticed that efficient financial practices could achieve a sustainable environment. Similarly, in the case of BRICS economies, Mngumi et al. [73] explored that financial development, technological innovations, and foreign direct investment have statistically significant inverse and long-run relationships with carbon emissions. Furthermore, Mensah and Abdul-Mumuni [74] found mixed results and argued that both positive and negative shocks in financial development lessen the carbon emissions in Sub-Saharan Africa. In another study, Tao et al. [75] demonstrated the significance of the non-linear nexus between financial development and carbon emissions intensity. Furthermore, Yang et al. [76] proved that financial intermediation has a more significant impact than financial development indicators in the 283 cities of China. Likewise, in China, Xiong et al. [77] noted the negative effect of financial development on environmental sustainability.

Following the existing literature, it is evident that studies in the field exhibit varying outcomes, even within the confines of a single country or region. This diversity in findings can be attributed to the utilization of disparate econometric methodologies and combinations of macroeconomic indicators. The divergence in results underscores the nuanced nature of economic analyses, emphasizing the impact of methodological choices on research outcomes. Considering these, it becomes important to recognize and contextualize the divergent results as an inherent consequence of the methodological heterogeneity prevalent in the examined studies. Consequently, future research endeavors, as done in this work, should remain cognizant of these methodological intricacies to enhance the coherence and comparability of findings in this domain.

## 3 Econometric model, data and methods

The current study attempts to develop the relationship between methane (CH_4_), a hydrocarbon component of natural gas, as a proxy of carbon emission, GDP as economic growth, financial developments (FIN), and medium and high technologies as a proxy of information technology (ICT) and human development (HDI). This study observes two extended moderating effect models of human development index and financial development via medium and high technologies on carbon emissions over the 15-year periods from 2007 to 2021 for the 27 EU economies. As explained previously, in the existing literature, several studies have discussed the positive and negative role of different economic indicators with multiple combinations, such as GDP, ICT, financial development, deforestation, Foreign Direct Investment (FDI) inwards and outwards, energy demands, renewable energy and non-renewable energy, fossil fuel, human development, natural resources, entrepreneurial activities, trade openness, tourism, etc., in the deterioration and amelioration of the environment quality around the Globe. For instance, some recent studies include Anwar et al. [78], Jabeen et al. [79], Chen et al. [80], Islam et al. [81], Bolat et al. [82], Kuzior et al. [83], Gronau et al. [84] Irfan et al. [85], Dahmani et al. [86], Kumari and Singh [87], Leitão et al. [26], Magazzino et al. [25], Jahanger et al. [27], Feng et al. [28], Mahmood et al. [30], Xing et al. [29], Wang et al. [33], Saia [31], Liu et al. [62], Bandy and Kocoglu [64], Jia et al. [88], Shobande et al. [89], Mngumi et al. [73] and You et al. [58]. Based on the existing literature we selected the econometric model for this study. Moreover, the current study adopted also the model from Chishti and Dogan [90] for the case of the top ten renewable energy user countries. Nonetheless, this study attempts to use different economic indicators and the moderating role of ICT to develop the relationships among the considered variables. The proposed models are as follows:

$${CH}_{4}=f\left(Economic\;Growth,Information\;technology,human\;development,financial\;development\;\right)$$

(Model 1) (1) $${CH}_{4it}=\gamma_{1it\;}+\gamma_{yit}{GDP}_{it}+\gamma_{dit}{ICT}_{it}+\gamma_{fit}{FIN}_{it}+\gamma_{hit\;}{HDI}_{it}+\epsilon_{it}$$

where CH_4_ is a regressand variable, whereas economic growth, information technology, financial development, and human development stand as regressors. Three models were constructed. In the first model (see equation (1)) the effect of emissions on considered regressors was analyzed. In the second (see equation (2)) and third models (see equation (3)), the interactions term was introduced to identify the moderating role of ICT and HDI, and ICT and FIN. The second and third econometric models are expressed as follows.

(Model 2) (2) $${CH}_{4it}=\gamma_{1it\;}+\gamma_{yit}{GDP}_{it}+\gamma_{dit}{ICT}_{it}+\gamma_{fit\;}{FIN}_{it}+\gamma_{hit\;}{HDI}_{it}+\gamma_{Idit}{ICT*HDI}_{it}+\epsilon_{it}$$

(Model 3) (3) $${CH}_{4it}=\gamma_{1it\;}+\gamma_{yit}{GDP}_{it}+\gamma_{dit}{ICT}_{it}+\gamma_{fit\;}{FIN}_{it}+\gamma_{hit\;}{HDI}_{it}+\gamma_{Ifit}{ICT*FIN}_{it}+\epsilon_{it}$$

We have that $\epsilon_{it}$ is an idiosyncratic error term, independent and identically distributed. It follows the usual assumption of a standard normal distribution with mean zero and constant variance. Whereas i represents the considered countries, t stands for a period, $\gamma_{1it\;}$ is the intercept, while $\gamma_{yit},\gamma_{dit},\gamma_{fit\;},\gamma_{hit\;}\gamma_{Ifit}$ are the long-run elasticity estimates of CH_4_ for the explanatory variables, such as GDP, ICT, FIN, HDI, and interaction terms, respectively.

### 3.1 Data

This study intends to examine the relationship among the economic indicators such as CH_4_, GDP, ICT, FIN, and HDI for 2007–2021 for the 27 European economies.^1^

The data for the considered economic indicators are taken from World Development Indicators [91] and the United Nations [92]. The periods and countries were selected based on the availability of such data. Descriptions, symbols, and supporting references are mentioned in Table 1.

**Table 1:** Variables used in this study descriptions.

Variables	Symbols	Definition	Supporting Reference	Source
Methane	CH_4_	Natural Logarithm of Methane emissions (kt of CO_2_ equivalent)	Adeel-Farooq et al. [93], Madaleno and Moutinho [41], Sun et al. [94], Islam et al. [81], Irfan et al. [85]	WDI
Information technology	ICT	Natural Logarithm of Medium and High-technology exports (% of manufactured exports)	Chishti and Dogan [90]	WDI
Financial development	FIN	Natural Logarithm of Domestic credit to private sector (% of GDP)	Anton and Afloarei Nucu (2020), Dahmani et al. [86], Kumari and Singh [87], Cheng et al. [95]	WDI
Economic Growth	GDP	Natural Logarithm of GDP per capita (constant 2015 US$)	Leitao et al. [26], Magazzino et al. [25], Jahanger et al. [27], Feng et al. [28], Mahmood et al. [30], Xing et al. [29], Wang et al. [33], AA Aloala et al. [96]	WDI
Human development	HDI	Human Development Index (HDI)	Kassouri and Altintas [97] Haini [98], Azam et al. [99], Liu et al. [62], Bandy and Kocoglu [64]	UNDP

**Fig. 1:**
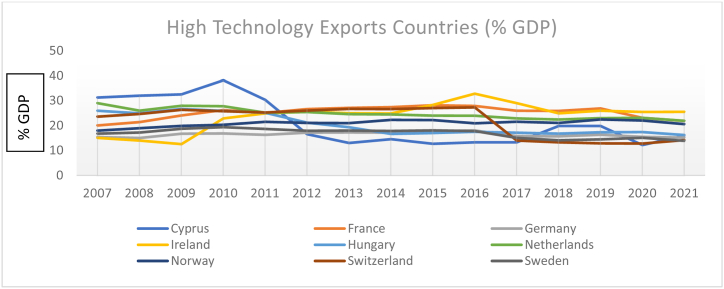
Country-level technology exports (% GDP).

**Fig. 2:**
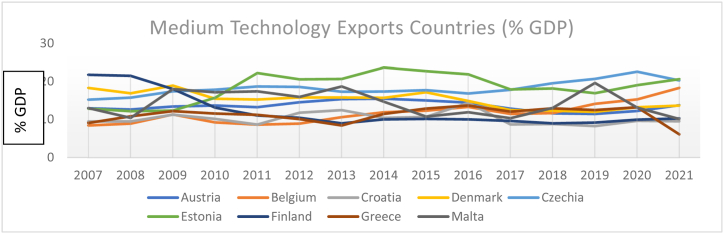
Country-level technology exports (% GDP).

**Fig. 3:**
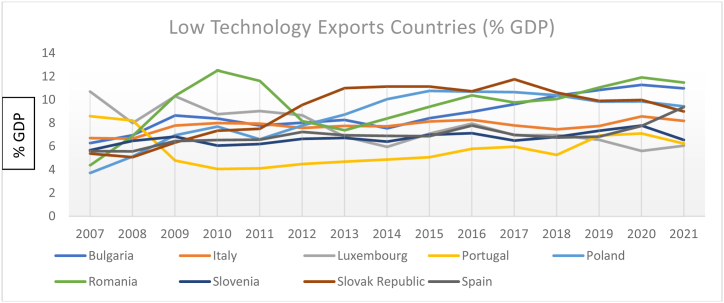
Country-level technology exports divided by high, medium, and low-level exporters (% GDP).

The country-level low, medium, and high-technology exports (% GDP) are presented in Fig. 1, Fig. 2, Fig. 3. Overall, medium, and high technology exports in the selected 27 EU economies have mixed trends during the research period. For instance, Malta, Cyprus, Switzerland, and Slovenia had a declining trend till 2016, and from 2016 to 2019, an increasing trend, and then again, a downward trend has been seen in 2021.

The results are expressed as total technology exports in % of GDP for each of the 27 EU Countries. We divided countries into three groups, low medium, and high based on average technology exports during 2007–2021. Fig. 1 represents the group of high-level technology export countries (average > 15 %); Fig. 2 represents the group of medium-level technology export countries average (10 %< and> 20 %); Fig. 3 represents the group of low-level technology export countries average (<10 %).

Sweden, France, Netherlands, Norway, Germany, and Portugal's results are consistent with their technology exports during the considered periods. Notably, Czech, Hungary, Ireland, and Belgium performed better than other EU-sampled countries during the considered research time concerning ICT high technology exports.

The summary of the considered variables of each sample country during the research period is described in Table 2. France, Netherlands, and Ireland are, on average, the leading exporters of ICT, whereas Luxembourg and Slovenia stand at the last position during the research period of 27 EU-selected countries.

**Table 2:** Country-wise descriptive statistics.

Countries	Var	Mean	SD	CV%	Countries	Mean	SD	CV%	Countries	Mean	SD	CV%
Austria	GDP	10.70	0.02	0.18	Germany	10.54	0.05	0.47	Croatia	9.44	0.08	0.84
	ICT	2.60	0.09	3.46		2.99	0.05	1.79		2.31	0.15	6.93
	HDI	0.90	0.07	7.77		0.92	0.00	1.08		0.83	0.01	1.20
	FIN	4.50	0.05	1.11		4.43	0.08	1.80		6.14	0.09	1.46
	CH_4_	8.93	0.06	0.67		10.88	0.09	0.82		8.22	0.01	0.12
Bulgaria	GDP	8.86	0.10	1.12	Greece	9.87	0.11	1.11	Cyprus	10.15	0.07	0.68
	ICT	2.16	0.17	7.87		2.40	0.21	8.75		2.96	0.41	13.85
	HDI	0.80	4.50	562		0.87	0.01	1.14		0.87	0.01	1.14
	FIN	4.05	0.01	0.24		4.57	0.20	4.37		5.23	0.34	6.50
	CH_4_	8.93	0.24	2.68		9.22	0.06	0.65		6.56	0.03	0.45
Belgium	GDP	10.61	0.02	0.18	Hungary	9.45	0.11	1.16	Czech	9.78	0.07	0.71
	ICT	2.43	0.22	9.05		2.99	0.19	6.35		2.89	0.10	3.46
	HDI	0.92	0.01	1.08		0.83	0.01	1.20		0.88	0.01	1.13
	FIN	4.14	0.10	2.41		3.77	0.24	6.36		3.88	0.08	2.06
	CH_4_	9.07	0.06	0.66		8.91	0.05	0.56		9.47	0.04	0.42
Denmark	GDP	10.89	0.04	0.36	Ireland	10.96	0.22	2.00	Estonia	9.77	0.12	1.22
	ICT	2.71	0.14	5.16		3.13	0.27	8.62		2.89	0.22	7.61
	HDI	0.93	0.01	1.07		0.91	0.01	1.09		0.87	0.01	1.14
	FIN	5.16	0.07	1.35		4.29	0.64	14.91		4.28	0.16	3.73
	CH_4_	9.06	0.07	0.77		9.63	0.04	0.41		7.06	0.07	0.99
Finland	GDP	10.70	0.03	0.28	Italy	10.35	0.04	0.38	Luxembourg	11.57	0.02	0.17
	ICT	2.45	0.30	12.24		2.04	0.06	2.94		2.02	0.19	9.40
	HDI	0.92	0.01	1.08		0.88	0.06	6.81		0.91	0.01	1.09
	FIN	4.51	0.07	1.55		4.44	0.07	1.57		4.55	0.10	2.19
	CH_4_	8.54	0.08	0.93		10.72	0.04	0.37		6.33	0.01	0.15
France	GDP	10.51	0.02	0.19	Netherlands	10.73	0.03	0.27	Malta	10.06	0.14	1.39
	ICT	3.22	0.10	3.10		3.20	0.08	2.5		2.61	0.25	6.92
	HDI	0.89	0.01	1.12		0.92	0.01	1.08		0.87	0.02	2.29
	FIN	4.60	0.09	1.95		4.70	0.05	1.06		4.54	0.19	4.18
	CH_4_	11.05	0.05	0.45		9.73	0.06	0.61		5.36	0.09	1.67
Norway	GDP	11.22	0.02	0.17	Portugal	9.89	0.04	0.40	Romania	9.11	0.14	1.53
	ICT	3.04	0.06	1.97		1.92	0.23	13.37		2.23	0.27	12.10
	HDI	0.89	0.22	24.71		0.84	0.01	1.19		0.81	0.01	1.23
	FIN	4.89	0.10	2.04		4.83	0.19	3.93		3.43	0.15	4.37
	CH_4_	8.44	0.05	0.59		9.36	0.02	0.21		9.75	0.05	0.51
Spain	GDP	10.16	0.04	0.39	Sweden	10.82	0.04	0.36	Slovenia	9.98	0.06	0.60
	ICT	1.99	0.12	6.21		2.92	0.11	3.90		1.89	0.07	3.70
	HDI	0.88	0.01	1.13		0.92	0.01	1.08		0.84	0.01	1.19
	FIN	4.88	0.23	4.71		4.84	0.05	1.03		4.06	0.28	6.89
	CH_4_	10.56	0.02	0.18		8.59	0.09	1.04		7.50	0.07	0.93
Switzerland	GDP	11.32	0.02	0.17	Poland	9.41	0.15	1.59	Slovakia	9.67	0.10	1.03
	ICT	3.03	0.32	10.56		2.10	0.30	14.28		2.17	0.27	12.44
	HDI	0.94	0.01	1.06		0.85	0.01	1.17		0.90	0.01	1.11
	FIN	5.10	0.06	1.17		3.90	0.09	2.30		3.93	0.18	4.58
	CH_4_	8.57	0.04	0.46		10.33	0.04	0.38		8.38	0.04	0.47

**Note:** The authors conducted an analysis of data from 27 European Union countries over the period 2007–2021, utilizing the natural logarithm. The results were summarized using several statistical measures: the mean (calculated as the simple average), the standard deviation (S.D.), and the coefficient of variation (CV).

In addition, the highest average methane emissions in the last fifteen years were observed in Germany, whereas the lowest ones were in Malta. Furthermore, the average methane in the EU region is 8.86, and exports of medium and high technology are 15.24 % of GDP (see Table 3). Likewise, the Human Development Index moves between 0.80 and 0.99 in these countries. Similarly, this study also noticed a coefficient variation (CV), which illustrates that various EU nations are consistent performers in their economic growth and human development. However, there is some variability over time in the case of ICT, methane, and financial development of EU countries.

**Table 3:** Overall countries descriptive statistics.

Variables	GDP	HDI	ICT	CH_4_	FIN
Mean	10.248	0.8866	15.245	8.8614	4.5099
Median	10.220	0.8920	13.146	8.9839	4.5312
Maximum	11.629	0.9620	53.018	11.141	6.2647
Minimum	8.7078	0.0940	3.7303	5.2312	3.2036
SD	0.6863	0.0578	8.2990	1.3712	0.5761
Skewness	−0.0674	−6.4645	1.3330	0.6393	0.4991
Kurtosis	2.2977	87.940	5.5458	3.1756	3.8287
Jarque-Bera	8.6283	124572	229.32	28.112	28.408
Probability	0.0133	0.0000	0.0000	0.0000	0.0000
Correlation Matrix					
GDP	1				
HDI	0.2771	1			
ICT	0.2931	0.1118	1		
CH4	−0.0760	0.0212	−0.1715	1	
FIN	0.3252	0.0631	0.1046	−0.1656	1

Note: The authors conducted an analysis of data from 27 European Union countries over the period 2007–2021, utilizing the natural logarithm. The results were summarized using several statistical measures: the mean (calculated as the simple average), the standard deviation (S.D.), and the coefficient of variation (CV).

Besides overall descriptive statistics for our variables of interest, correlation coefficients are reported in Table 3. The results show that methane has a weak negative correlation with economic growth, ICT, and financial development in 27 EU countries, whereas the rest of the variables directly relate to each other.

### 3.2 Methodology

The present research endeavors to discern the impact of macroeconomic variables, including Information Technology (ICT), Financial Development (FIN), Human Development (HDI), and Economic Growth (GDP), on climate change mitigation. Additionally, this work delves into the moderating influence of ICT in the realms of human development and financial development regarding methane emissions intensity. For this purpose, this study used a two-step method approach to address this problem. The first step is the standard econometric techniques, methods, and tests, and the second one is machine learning (ML) modeling, nowadays prevailing as a new trend, and seen with some excitement among researchers, to forecast methane (CH_4_) emissions.

#### 3.2.1 Econometric methods and techniques

The current study aims to detect the short and long-run relationship among the considered variables. Moreover, the study intends to highlight the moderating role among the variables. Furthermore, this research work also tries to explore the causal relationship among the economic indicators from 2007 to 2021 in the 27 EU economies.

The world has become a global village, especially using ICT, digital marketing and other communication mediums, social media applications, and many more integration and unions stand nations on the same page. Unequal population distribution, country classification based on income, labor shortage, and surplus, aging gaps, and scattered natural resources made nations interdependent. However, handling climatic changes and upcoming economic challenges to achieve the sustainable economic goals suggested by the United Nations by 2030 encourages countries to stand together and the actions of one impacts the outcomes of all. Thus, global economies are interdependent, and this is especially valid for EU countries, which seem to be more interlinked than the rest of the World. Econometricians name this interdependence cross-sectional dependence (CSD) and claim that applying the traditional method in the presence of CSD may produce spurious and biased results (Chishti and Sinha, 2022).

The current study deploys various cross-sectional tests to identify the potential CSD across the modeled series such as Pesaran [100] recognized as CD; Juodis and Reese [101] identified as CDw; Fan et al. [102] turned it known as CDw+; and Pesaran and Xie [103] used notation is as CD*. In addition, this research work also employs the slope heterogeneity test among the series to verify the heterogeneity, and for exponent, cross-sectional exponent tests are executed. Hereafter, the next step was to examine the stationary characteristics of each selected variable. The stationary process is testified by applying second-generation cross-sectional Augmented Dickey-Fuller unit root and augmented cross-sectional panel unit root tests such as CIPS and CADF introduced by Pesaran [104]. In addition, this study also applied the third-generation Karavias and Tzavalis panel unit root with a structural break [105] to identify the stationary process.

The next step after the confirmation of the CSD and the stationary process is to check the cointegration among the variables. For this purpose, the study used the first--, second-, and third-generation cointegration tests to find the existence of a long-run relationship between the sample variables. The first-generation cointegration test was introduced by Pedroni [106] to identify the stationary process. Pedroni cointegration is the residuals-based test that allows both short-run dynamics and long-run slope coefficients to be heterogeneous across individual members of the panel. The tests consider both pooled within-dimension tests and group mean between-dimension tests, and they also reflect individual heterogeneous fixed effects and trend terms (Pedroni, 2004). Furthermore, the second-generation Westerlund [107] panel cointegration test, which is appropriate in the presence of CSD, was then deployed. In addition, the most recent panel cointegration test by Westerlund and Edgerton [108] which is suitable for the dependent panels in the presence of structural breaks was also employed.

Recent research work applies the auto-regressive distributed lag model cross-sectional (ARDL-CS) in the presence of CSD, heterogeneity, and mixture of integrated order such as I (0) and I (1) to detect the short and long-run coefficients. It can provide robust results, unlikely traditional Auto Regressive Distributed Lag model (ARDL), Fully Modified Ordinary Least Squares (FMOLS), and Dynamic Ordinary Least Squares (DOLS) methods. Moreover, it is also suitable to tackle autocorrelation, heteroscedasticity, and endogeneity in the sample series [90]. This methodology was therefore used in the presented study herein. Lastly, in the econometric methodology section, the causal nexus between the considered variables by Dumitrescu and Hurlin's [109] panel causality test was also explored. This is suitable for the balanced and heterogeneous panel series used and presents an innovative approach to the set of variables selected for this purpose.

#### 3.2.2 Machine learning algorithms

Recent research adopts the machine learning algorithm to predict each sampled country's future value of methane (CH_4_). This analysis study implemented and compared eight different machine learning maximum likelihood (ML) algorithms such as Simple Regression Tree (LRT), Polynomial Regression (PR), Random Forest Regression (RFR), Probabilistic Neural Networks (PNN), Artificial Neural Networks (ANN), Gradient Boosted Tree (GBT), Linear Regression (LR) and Tree Ensemble Regression (TER). The algorithm is judged based on its ability to maximize the goodness of fit (R-squared) (see equation (4)). Specifically, the following definitions have been used:

(4) $$R^{2}=\frac{Explained\;sum\;of\;square}{Total\;sum\;of\;squares}=1-\frac{Sum\;of\;the\;Squared\;of\;residuals}{TotalSumOfSquares}=1-\frac{\sum {\left(y_{i}-{\hat{y}}_{i}\right)}^{2}}{\sum {\left(y_{i}-{\bar{y}}_{i}\right)}^{2}}$$

and minimize statistical errors such as.

Mean Standard Error (MSE) (see equation (5))

(5) $$MSE=\frac{1}{n}\sum_{i=1}^{n}{\left(Y_{i}-\hat{Y_{i}}\right)}^{2}$$

Mean Average Error (MAE) (see equation (6))

(6) $$MAE=\frac{\sum_{i=1\;}^{n}\left|y_{i}-x_{i}\right|}{n}=\frac{\sum_{i=1}^{n}\left|e_{i}\right|}{n}$$

Mean Signed Difference (MSD) (see equation (7)) or Mean Signed deviation or Mean Signed Error

(7) $$MSD(\hat{\theta )}=\frac{1}{n}\sum_{i=1}^{n}\hat{\theta_{i}}-\theta_{i}$$

and Root Mean Square Error (RMSE) (see equation (8)):

(8) $$RMSE=\sqrt{\frac{1}{N}\sum_{i=1}^{N}{\left(\hat{y_{i}}-y_{i}\right)}^{2}}$$

Furthermore, this study ranked the different algorithms based on maximum R^2^ and minimum MSE, MAE, MSD, and RMSE and declared the best one to forecast the value of methane emissions. Afterward, an aggregate ranking was created, merging the orders on each indicator. The method with the lowest score was chosen since it ranks highest among each indicator. The sample was divided into two parts, with 30 % of the data used for testing and 70 % for the training set.

## 4 Results and discussion

### 4.1 Econometric results and discussion

The current research analyzed the effect of GDP, ICT, FIN, and HDI on Methane as a proxy of carbon emissions in the sampled 27 EU economies. Moreover, the current study also explored the dual impact of ICT and HDI, and ICT and FIN on carbon emissions for 2007–2021 within the EU space.

The results of the econometric analysis have demonstrated the following outputs. Firstly, the study applied multiple first and second-generation cross-sectional dependence (CD) tests to identify the interdependence between the sample countries. In the initial step, Pesaran's [100] CD test was applied, whereas a 1 % level of significant values in the first column of Table 4 shows that rejecting the null hypothesis of weak cross-sectional dependence and acceptance of the alternative hypothesis of strong cross-sectional dependence ensues among the 27 EU economies. Additionally, the study also confirmed cross-sectional dependency with other CD tests such as Juodis and Reese [101], recognized as CDw, Fan et al. [102], known as CDw+, and Pesaran and Xie [103] symbolization as CD* for the robustness of the Pesaran [100] findings.

**Table 4:** Cross-sectional dependence, slope heterogeneity, and exponent estimations tests.

Tests	CD			CD_w_	CD_w+_	CD*
Variables	T-stat	Corr	Abs corr	T-stat	T-stat	T-stat
GDP	43.35^a^ (0.00)	0.597	0.674	2.76^a^ (0.00)	901.93^a^ (0.00)	−2.01^a^ (0.00)
HDI	59.86^a^ (0.00)	0.82	0.826	−0.41^a^ (0.00)	1113.86^a^ (0.00)	1.97 ^b^ (0.03)
ICT	2.91^a^ (0.00)	0.04	0.479	−0.32^a^ (0.00)	613.69^a^ (0.00)	0.622^c^ (0.10)
CH_4_	37.35^a^ (0.00)	0.515	0.681	−1.72^a^ (0.00)	900.50^a^ (0.00)	−1.50^c^ (0.10)
FIN	5.72^a^ (0.00)	0.079	0.676	0.085^a^ (0.00)	906.62^a^ (0.00)	0.69 (0.493)

Estimation of Cross-Sectional Exponent (alpha)				Slope heterogeneity tests		
	Alpha	[95 % Conf. Interval]			Δ	$\Delta_{\text{Ajusted}}$
GDP	0.98	0.90	1.06	Model 1	13.187^a^ (0.00)	17.024^a^ (0.00)
HDI	1.01	0.90	1.12	Model 2	10.18^a^ (0.00)	13.95^a^ (0.00)
ICT	0.73	0.65	0.80	Model 3	11.015^a^ (0.00)	15.083^a^ (0.00)
CH_4_	0.94	0.63	1.26			
FIN	0.99	0.95	1.03			

Note: The *P*-values are reported in the parathesis. Whereas a, b, and c represent 1 %, 5 %, and 10 % levels of significance. Moreover, in the case of the cross-sectional exponent. 0.5 ≤ alpha <1 implies strong cross-sectional dependence. Variables are centered around zero.

The second step estimation of the cross-sectional exponent also reconfirmed the strong presence of the cross-sectional dependence. Though cross-sectional dependence was found in the study, the latest techniques to improve the efficacy of the study were employed. Furthermore, the study also found that the slopes of all the considered models are heterogeneous rather than homogenous. According to Park et al. [110], the panel data was gathered by grouping cross-sections from various institutional setups, cultural distinctions, and unique characteristics of each country. As a result, the CD has more possibilities, which could lead to biased and spurious results. These results are in line with those of Saqib et al. [23] and Madaleno and Moutinho [41] for EU countries. Secondly, multiple second-generation unit root tests were employed to identify the stationary process.

There is a need to use more than the traditional unit root Augmented Dickey-Fuller (ADF) test, especially in the presence of cross-sectional dependence. Consequently, the two well-known second-generation unit root tests, such as CIPS and CADF, were employed to examine the stationary process. Additionally, the current study also applied the Karavias and Tzavalis panel unit root with a structural break introduced by Chen et al. [105]. The unit root results are reported in Table 5, indicating that most variables are stationary at first difference. Structural breaks were also identified in the selected countries panel. The incidence of structural breaks in 2013 in the panel of the sampled countries is very clear. For example, the sample economies were devastated by multiple financial crises, such as the 2008 global crisis and the 2010 European financial crisis and this is observed in the reported results of this study's analysis.

**Table 5:** Unit root tests.

Second-Generation Unit root tests			Karavias and Tzavalis structural break		
Variables	CIPS	CADF	Statistic	Break	CD
GDP	−3.18^a^	−1.67	−4.47	2013	yes
ΔGDP	−2.99	−2.69^a^	−11.59^a^	2013	yes
ICT	−1.73	−1.63	−0.93	2013	yes
ΔICT	−2.95^a^	−2.16^a^	−12.02^a^	2013	yes
FIN	−1.55	−1.91	1.49	2013	yes
ΔFIN	−2.78^a^	−2.50^a^	−8.72^a^	2013	yes
HDI	−2.19	−3.02	−15.87^a^	2013	yes
ΔHDI	−3.49^a^	−4.37^a^	−19.96^a^	2013	yes
CH_4_	−2.07	−1.32	−6.67^a^	2013	yes
ΔCH_4_	−3.35^a^	−2.41^a^	−14.46^a^	2013	yes

Note: a, b, and c represent 1 %, 5 %, and 10 % levels of significance.

The next objective was to detect the long-run relationship among the considered variables. Therefore various cointegration tests, named Pedroni [111], and Westerlund [107], were employed. The cointegration results are presented in Table 6, which confirms that the long-run relationships exist among the considered variables. It means that the sampled variables move together in the long run. Similar results were reported by previous authors [39,40,112].

**Table 6:** Panel cointegration tests.

Westerlund		Pedroni			
	Statistic		Model 1	Model 2	Model 3
Model 1	1.52^c^ (0.06)	Modified Phillips–Perron t	6.30^a^ (0.00)	7.41^a^ (0.00)	7.50^a^ (0.00)
Model 2	2.08^a^ (0.01)	Phillips–Perron t	−3.65^a^ (0.00)	−5.18^a^ (0.00)	−3.41^a^ (0.00)
Model 3	2.43^a^ (0.00)	Augmented Dickey-Fuller t	3.55^a^ (0.00)	−4.28^a^ (0.00)	−3.92^a^ (0.00)

Westerlund and Edgerton						
	$Z_{\emptyset }\left(N\right)$			$Z_{\tau }\left(N\right)$		
	No break	Mean shift	Regime shift	No break	Mean shift	Regime shift
Model 1	−3.40^a^	−3.28^a^	−3.15^a^	−3.50^a^	−3.38^a^	−3.20^a^
Model 2	−3.80^a^	−3.71^a^	−3.61^a^	−3.70^a^	−3.63^a^	−3.41^a^
Model 3	−3.39^a^	−3.25^a^	−3.19^a^	−3.49^a^	−3.39^a^	−3.25^a^

Note: H0: No co-integration and Ha: All panels are co-integrated. Whereas p-values reported in parentheses.

By utilizing the Westerlund and Edgerton [108] test the credibility of the cointegration process was improved. However, Westerlund and Pedroni cointegration tests may produce spurious results in the presence of a structural break.

After confirming the cointegration among the variables of the sampled 27 economies of the EU, the dynamic auto-regressive distributed lag-cross-sectional (ARDL-CS) technique was employed to identify the short and long-run coefficients. Table 7 depicts the results of all the considered models for this study. The first used model results show that economic growth, information technology, and human development improved the environment in the 27 panels of EU economies. On the contrary, financial development deteriorated the environment quality in the sampled countries for 2007–2021. However, economic activities and financial development lessened, and human development improved the environmental quality in the short run. Moreover, the error correction term also confirms that all cross-sections (study countries) in the long run are moving aligned. These results align with those of previous authors, such as Wiredu et al. [72], Tao et al. [75], and Mensah and Abdul-Mumuni [74].

**Table 7:** ARDL-CS results.

Dependent variable: CH_4_						
Methods	Model 1		Model 2		Model 3	
Variables	Coefficient	Prob	Coefficient	Prob	Coefficient	Prob
Long-run coefficients						
GDP	−0.18^a^	0.00	−0.24^a^	0.00	−0.22^a^	0.00
FIN	0.05^a^	0.00	0.06^a^	0.00	0.10^a^	0.00
ICT	−0.05^a^	0.00	−0.10^a^	0.00	−0.11^a^	0.00
HDI	−1.20^a^	0.00	−1.16^a^	0.09	−0.98^c^	0.06
ICT*HDI			−1.48	0.00		
ICT*FIN					−0.32^a^	0.00
Error correction coefficients	−0.16^a^	0.01	−0.22	0.00	−0.17^a^	0.00
Short-run coefficients						
D(GDP)	0.16^a^	0.00	0.02^c^	0.09	0.10^c^	0.10
D (FIN)	0.09^a^	0.03	0.03 ^b^	0.04	0.06^c^	0.08
D(ICT)	0.01	0.43	0.05	0.36	0.10 ^b^	0.05
D(HDI)	−0.12	0.78	−0.14	0.43	−0.05 ^b^	0.06
D(ICT*HDI)			1.80	0.32		
D(ICT*FIN)					−1.06^c^	0.10
C	3.41^a^	0.00	2.60^a^	0.00	2.20^a^	0.00

Note: a, b, and c represent 1 %, 5 %, and 10 % levels of significance.

The second model considered the moderating effect of ICT and HDI on carbon emissions. The results demonstrate that the moderating effect of ICT with HDI also improved environmental sustainability. It means that using information technology in human development is a positive measure, which may not only lessen carbon emissions but also positively contribute to human development and economic efficacy. Furthermore, considering the results of ARDL-CS estimates for the case of model three, results show that the moderating role of ICT with financial activities has some positive impact on climatic changes. These results are a line with those of previous and recent research [76,90].

If the ARDL-CS estimates are summarized, the results show that economic growth in model 1 (18 %), model 2 (24 %), and model 3 (22 %) have a mild positive effect on carbon emissions. Using information technology in human development and financial activities has resulted in a more sustainable effect on the environment. Similarly, for the case of ICT, HDI, and financial development, the results remarked that dual performance is better than the individual role of ICT, HDI, and financial development (see model 2 and model 3 results).

Finally, the causality analysis results in Table 8 demonstrate the causal relationships among the selected variables in the 27 European sampled countries. The 3rd, 5th^,^ and 7th columns of the table indicate the direction of the causality for each variable.

**Table 8:** DH-causality test results.

Null Hypothesis	Model 1		Model 2		Model 3	
	W-Stat.	Causality	W-Stat	Causality	W-Stat	Causality
GDP — CH_4_	6.28^a^	GDP↔ CH_4_				
CH4—GDP	9.92^a^					
FIN— CH_4_	5.30^a^	FIN↔ CH_4_				
CH4—FIN	6.79^a^					
ICT— CH_4_	3.40	ICT—CH_4_				
CH4—ICT	4.99^a^	CH_4_→ ICT				
HDI— CH_4_	4.55^a^	HDI↔ CH_4_				
CH_4_—ICT	5.30^a^					
FIN—GDP	9.42^a^	GDP↔ FIN				
GDP—FIN	10.47^a^					
ICT—GDP	4.62^a^	GDP↔ICT				
GDP—ICT	4.88^a^					
HDI—GDP	9.51^a^	GDP↔ HDI				
GDP—HDI	5.17^a^					
ICT—FIN	4.14^a^	FIN↔ ICT				
FIN—ICT	6.76^a^					
HDI—FIN	6.73^a^	HDI↔ FIN				
FIN—HDI	5.13^a^					
HDI—ICT	6.47^a^	HDI→ ICT				
ICT—FIN	3.50	ICT—FIN				
ICT*HDI—CH4			3.08	ICT*HDI—CH_4_		
CH4 — ICT*HDI			4.99^a^	CH_4_→ ICT*HDI		
ICT*HDI—GDP			4.51^a^	GDP↔ ICT*HDI		
GDP — ICT*HDI			5.42^a^			
ICT*HDI—FIN			4.67^a^	FIN↔ ICT*HDI		
FIN — ICT*HDI			7.15^a^			
ICT*FIN—CH4					4.34^a^	CH_4_↔ ICT*FIN
CH4 — ICT*FIN					4.80^a^	
ICT*FIN—GDP					4.95^a^	GDP↔ ICT*FIN
GDP — ICT*FIN					4.52^a^	
ICT*FIN—HDI					4.60^a^	HDI↔ ICT*FIN
HDI — ICT*FIN					5.78^a^	

Note: The results are presented with the use of symbols, where ↔, →, — denote that the values are insignificant and indicate bidirectional, unidirectional, or no causality respectively. Whereas a, b, and c represent 1 %, 5 %, and 10 % level of significance.

The results show four significant unidirectional causalities (see Table 8) running from CH_4_ to ICT, HDI to Information Technology (ICT), and CH_4_ to ICT*HDI. Additionally, the study noted bidirectional causality between FIN and CH_4_, GDP and CH_4,_ HDI and CH_4,_ GDP and FIN, GDP and ICT, GDP and HDI, FIN and ICT, FIN and HDI, GDP and ICT*HDI, FIN and ICT*HDI, GDP and ICT*FIN, CH_4_ and ICT*FIN, and HDI and ICT*FIN. However, no causality was discovered between ICT and CH_4_, ICT and FIN, and ICT*HDI and CH_4_, in European Union countries from 2007 to 2021.

### 4.2 Machine learning results and discussion

**Fig. 4:**
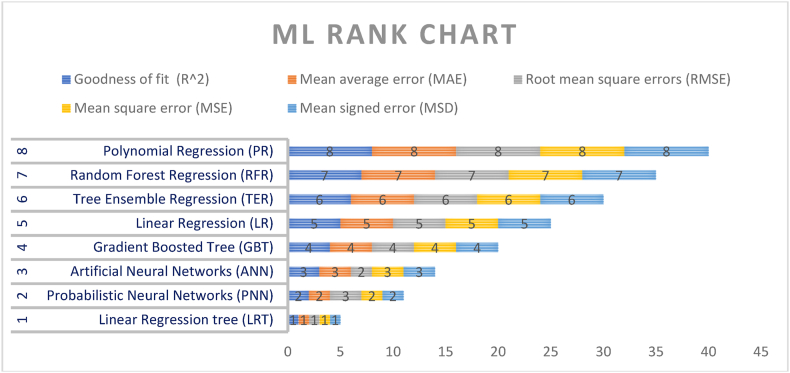
Machine learning algorithm overall ranking chart (Note: Authors elaborations in Flourish studio).

The results from Fig. 4 clearly show how the overall ranking is almost identical to the single orders produced by each algorithm. The following ordering of the applied algorithms is obtained in Table 9. Moreover, the scores obtained by each algorithm such as Probabilistic Neural Networks (PNN), Artificial Neural Networks (ANN), Gradient Boosted Tree (GBT), Simple Regression Tree (LRT), Polynomial Regression (PR), Random Forest Regression (RFR), Linear Regression (LR), and Tree Ensemble Regression (TER) for the single statistical indicator are presented in Fig. 4.

**Table 9:**
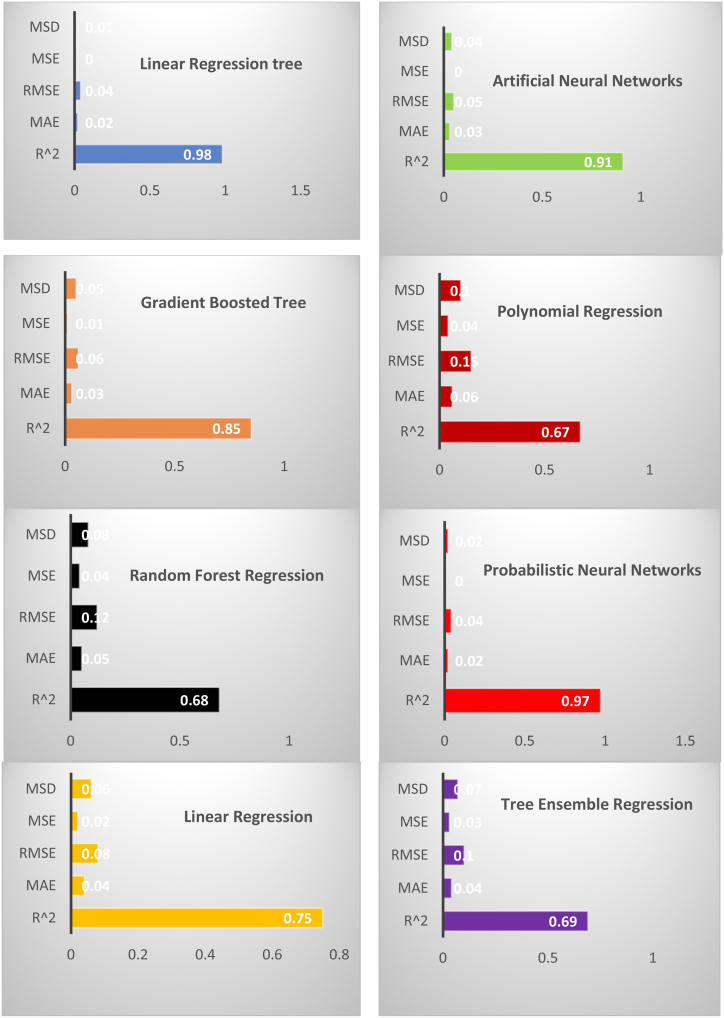
Statical performance of ML algorithms.

Using rankings, payoff scores (sum of the errors ranking), statistical errors, and goodness of fit (R-squared), the linear regression tree (LRT) is the best algorithm to predict the methane of each country based on first-ranked, minimum payoff score, low statical errors, and high R-squared.

**Fig. 5:**
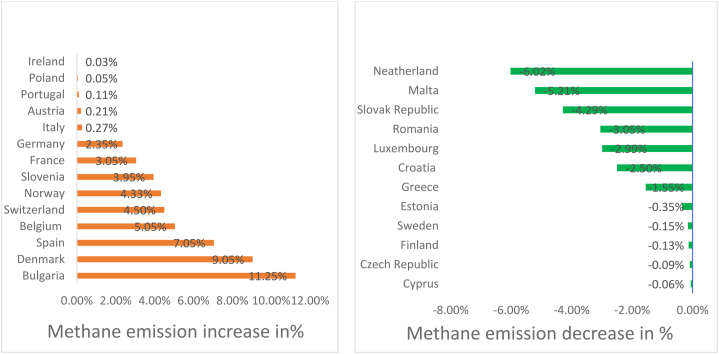
Methane predictions (Note: Authors elaborations in Flourish studio).

The STR model workflow comprises three components to declare as the best predictor algorithm: data preparation, machine learning and predictions, and the scores of the statistical errors. Moreover, through the application of the STR model, it is possible to classify both the green and yellow countries. Green and yellow countries represent the countries for which a reduction and increase, respectively, in the value of methane emissions is predicted after three years (see Fig. 5). Furthermore, in the case of the entire panel, the STR algorithm predicts an average growth in methane emissions of around 3.64 %.

**Green Countries:** The current research work with the Machine learning STR algorithm process points out that the Netherlands (6.02 %), Malta (−5.21 %), Slovak Republic (−4.29 %), Romania (−3.05 %), Luxembourg (−2.99 %), Croatia (−2.50 %) and Greece (−1.55 %), are the countries for which a significant reduction in the value of methane emissions is predicted. In addition, the study found there is a positive expectation, but less than one percent change, in Estonia, Sweden, Finland, Czech Republic, and Cyprus (see Fig. 5).

**Yellow Countries:** Furthermore, the so-called yellow countries where significant growth in methane emissions is predicted after three years are Bulgaria (+11.25 %), Denmark (+9.05 %), Spain (+7.05 %), Belgium (+5.05 %), Switzerland (+4.50 %), Norway (+4.33 %), Slovenia (+3.95 %), France (3.05 %). In contrast, Italy, Austria, Portugal, Poland, and Ireland have the no change result associated.

## 5 Policy implications

Climate change and sustainable growth are important for the future on a global scale. Considering the available existing literature, mixed impacts of digitalization and ICT over sustainable development and climate change mitigation are found. Usually, ICT and digitalization are found to improve environmental sustainability in several studies [2] through their mitigation of carbon emissions. However, ICT's potential may be questioned regarding its negative contribution to GHG emissions [113]. Even so, most of the literature accounts for climate and sustainable environmental proxies using carbon emissions, even if other GHG components, such as methane emissions are relevant, are still unexplored. Moreover, empirical methodologies and econometric models used lack sufficient justification [2], being traditional panel models used for this assessment, and powerful tools such as machine learning algorithms are ignored in the literature. This is a caveat that this study aimed to address.

Economic growth, information technology, and human development improved the environment in the 27 panels of EU economies. Green growth means expanding without harming environmental health. Therefore, these results point out that the 27 EU countries are on the correct path in terms of economic growth and respecting the EKC hypothesis in general. This is mostly a reflection of major key policies and strategies in the EU that foster a sustainable and greener future, such as the EU Green Deal, or the Fit for 55 to name a few. As such, this study supports these policy frameworks and reinforces the recommendation to increase the integration and implementation at the national, regional, and local levels of such green and sustainable frameworks and incentives. Linked to these frameworks seems also to be the digitalization development and human development stages within the sampled countries. Lange et al. [113] provide a list of four insights from ecological economics: (a) physical capital and energy are complements in the ICT sector, (b) increases in energy efficiency lead to rebound effects, (c) ICT cannot solve the difficulty of decoupling economic growth from exergy, (d) ICT services are relatively energy intensive and come on top of former production [113]. The authors even argue that digitalization can only boost sustainability when it fosters effects b) and d), without promoting effects a) and c). Considering that higher energy efficiency and the adoption of green technologies could soften the effect of ICT on CO_2_ emissions [114], the 27 EU countries, provided the present results, seem to be on the right path in this regard. This is also reasonable to happen considering their development stage and the EU legislation which demands for climate change reduction measures to be implemented in national legislations and supports clear ambitions and goals to be reached by 2030 and 2050. With the recent conflicts happening (e.g. the Ukraine-Russia and the Israel-Palestine conflicts), the energy transition processes are changing, and EU countries are ever more focused on strategic autonomy and reducing fossil fuels consumption, heavily incentivizing an increase in renewable energy production and consumption. This can only be done at the expense of digitalization and innovative green technologies. Thus, these results seem to follow the conclusions of previous works as to the positive effect of ICT on emissions, reducing them [49,50,[55], [56], [57]], contributing to sustainable development and climate change mitigation. On the contrary, the results herein presented contradict those of Uddin et al. [59] proving that ICT deteriorates environmental quality, and partially agree with Shobande and Asongu [51] which found mixed evidence.

As expected, human development seems to improve the environment in the sampled countries. As industries evolve, they'll increasingly focus on cleaner processes and technologies, foster service roles, limit pollution, and consume fewer resources. This progression could be boosted by government spending on health, education, and overall societal skills development. A well-educated workforce, coupled with greater earnings, is crucial for embracing sustainable consumption and production methods [60,[65], [66], [67]]. Our findings underscore the priority of advancing human development as the cornerstone of global development strategies, emphasizing that enhancing human welfare is crucial for fostering sustainable progress. Involvement from industrialized nations, as is the case of the 27 EU countries, aligned with the Sustainable Development Goals' broad agenda, is a crucial prerequisite for fostering development. The globalization phenomenon holds the potential for tremendous benefits for emerging economies, provided they are equipped with the proper tools to steer this process toward bolstering human potential through improved health and education standards, and developed economies have a crucial role in this. Elevating technological competency can shift these economies from relying heavily on natural resource depletion to becoming more knowledge-centric economies, curbing the overuse and harm to the environment, and promoting a positive loop of economic and human growth. Thus, the results herein presented do not conform to the view that human development increases emissions [62,64], damaging the environment and sustainable growth and development, and even being a country group analysis mixed results in this relationship were not found, as did Glennerster and Jayachandran [61].

The financial system can enhance environmental improvements through the efficient allocation of resources [69,115]. Sustainable financial practices help to embrace sustainable development [71,72]. However, the results obtained in this study seem to point out that financial development somehow deteriorated the environmental quality in the sampled countries for 2007–2021, although economic activities and financial development together lessened, and human development improved the environmental quality in the short run. Thus, results seem to suggest a better, or more directed, development of the financial system, as green finance developments, are needed to achieve environmental quality in the 27 EU countries. This negative effect of financial development on the environment confirms the findings of Ren et al. [69] and Xiong et al. [77], contradicting those finding a direct positive effect [70,72], or even those that point to mixed effects [69,71,74].

One of the contributions of the present work was the consideration of the interaction effect between ICT and HDI, and ICT and FD. Integrating Information Technology (ICT) with the Human Development Index (HDI) was observed to, not only, enhance environmental sustainability, but also, aid in reducing greenhouse gas emissions, bolstering human development, and strengthening economic efficiency. Additionally, evidence from ARDL Cointegration System analysis for the third model used indicates that the interplay between ICT and financial activities favorably affects climate change mitigation. This synergy between ICT, HDI, and financial development proves to be more effective in promoting environmental health than each element functioning alone, as demonstrated by the outcomes in the second and third models.

The STR model's process involved three key steps for identifying the most effective prediction method: preparing data, employing machine learning techniques, and generating forecasts along with calculating statistical error rates. The STR model also enabled the classification of countries into two categories: “green” countries expected to lower methane emissions and “yellow” countries where an increase is anticipated over the next three years (refer to Fig. 4). Additionally, when examining the entire dataset, the STR model anticipated an average escalation in methane emissions of about 3.64 %. The set of detected yellow countries is those where improvements in HDI, sustainable economic growth, ICT, and FD are required, namely Bulgaria, Denmark, Spain, Belgium, Switzerland, Norway, Slovenia, and France. In contrast, Italy, Austria, Portugal, Poland, and Ireland have the no change result associated. The Netherlands, Malta, Slovak Republic, Romania, Luxembourg, Croatia, and Greece, are the countries for which a significant reduction in the value of methane emissions is predicted, already following the right path in sustainable development and climate change mitigation.

## 6 Conclusion

Considering that machine learning algorithms are trained to find relationships and patterns in data, the current research analyzed the effect of GDP, ICT, FIN, and HDI on Methane as a proxy of carbon emissions in the sampled 27 EU economies, including the dual impact of ICT and HDI, and ICT and FIN on climate change for 2007–2021. This article starts by applying traditional ARDL-CS methods (multiple first and second-generation cross-sectional dependence (CD) tests to identify the interdependence between the sample countries), further using machine learning algorithms to identify green and yellow 27 EU countries groups, i.e., the countries for which a reduction and increase, respectively, in the value of methane emissions is predicted after three years as a tool to evaluate current and needed policy frameworks for sustainable development actions.

Results point that ICT, economic growth, and HDI improve environmental quality and contribute to climate change mitigation, through methane emissions reduction in the 27 EU countries. However, considering the individual effect of financial development on environmental quality, results show that it may damage the environment, by increasing emissions. Considering this, the joint crossed effects of ICT with HDI, and that of ICT with FD, were considered in estimations, with results pointing out that those combinations favorably affect climate change mitigation efforts and results. Therefore, this synergy between ICT, HDI, and financial development proves to be more effective in promoting overall EU environmental health than each element functioning alone, as expected. Policymakers in EU countries should bear this in mind when promoting the use of ICT, namely in financial markets, promoting the use of green financing sources and models to evaluate and select investments, when implementing green technologies. Furthermore, enhancing renewables production and consumption, having in mind the need to have adequate human capital able to deal with all these required changes, is of uttermost importance too. Education and general literacy in green and green financial development and enhancement (in both quantity and quality) could be a future strategy to be followed contributing to increased sustainable development.

While the primary aim of this study was to investigate the potential ramifications of digitalization, human development, and financial development on carbon emissions, there are discernible gaps in the analysis that can serve as guiding principles for future research. First and foremost, the multifaceted nature of digitalization poses a challenge when assessing its development stage due to the lack of comprehensive indicators. Consequently, the establishment of a more intricate indicator system is imperative to create robust analysis and results. To gain deeper insights into how the digital economy indirectly influences carbon emissions and incentivize nations to leverage the digital economy for carbon emissions reduction, a comprehensive analysis of the transmission mechanisms is warranted. Moreover, it is important to note that this study restricts its focus to the European Union (EU) when examining the impact of digitalization on carbon emissions, when it is commonly accepted that the world is interconnected, and the actions of one's impact the lives of all in the planet. To further enrich this research, future studies should expand their scope to encompass diverse regions across the globe and explore the variations in the digitalization influence on carbon emissions in these areas.

## Funding

We acknowledge funding from the BESIDE project (Horizon 2020 Coordination and Support Actions (CSA); Grant agreement ID: 951389) DOI: 10.3030/951389, funded by the 10.13039/501100000780European Commission.

We acknowledge also financial support to 10.13039/100013239CESAM by FCT/MCTES (UIDP/50017/2020+UIDB/50017/2020+LA/P/0094/2020), through national funds. We also thank the support of the Research Unit on Governance, Competitiveness and Public Policies (UIDB/04058/2020)+(UIDP/04058/2020) funded by national funds through 10.13039/501100001871FCT – Fundação para a Ciência e a Tecnologia.

## Ethics approval and consent to participate

Not applicable.

## Consent for publication

Not applicable.

## Data availability

The study has adopted data from the World Development Indicators (WDI) database and from the United Nations Development Program (UNDP).

## CRediT authorship contribution statement

**Zeeshan Arshad:** Writing – original draft. **Mara Madaleno:** Formal analysis, Data curation, Conceptualization. **Ana I. Lillebø:** Writing – review & editing, Funding acquisition. **Helena Vieira:** Writing – review & editing, Supervision.

## Declaration of competing interest

The authors declare that they have no known competing financial interests or personal relationships that could have appeared to influence the work reported in this paper.

## Footnotes

1 Austria, Belgium, Bulgaria, Croatia, Cyprus, Czech Republic, Denmark, Estonia, Finland, France, Germany, Greece, Hungary, Ireland, Italy, Luxembourg, Malta, Netherlands, Norway, Poland, Portugal, Romania, Slovenia, Slovak Republic, Spain, Sweden, Switzerland.
